# *Annona muricata* Leaf Extract Rebalances Systemic Redox Status and Modulates Neuroimmune, Microbial, and Functional Responses in CLP-Induced Septic Rats

**DOI:** 10.3390/antiox15101297

**Published:** 2026-10-07

**Authors:** Ufuk Ülker, Bülent Bayraktar, Mehmet Eray Alçığır, Sibel Kızıl, Fatih Aydin, Gökşad Cemil Kotan, Mustafa Yeni

**Affiliations:** 1 Department of Veterinary Microbiology, Department of Preclinical Sciences, Faculty of Veterinary Medicine, Yozgat Bozok University, 66100 Yozgat, Türkiye; ufuk.ulker@bozok.edu.tr; 2 Department of Physiotherapy and Rehabilitation, Faculty of Health Sciences, Bayburt University, 69000 Bayburt, Türkiye; bulentbayraktar@bayburt.edu.tr; 3 Department of Veterinary Pathology, Department of Preclinical Sciences, Faculty of Veterinary Medicine, Kırıkkale University, 71450 Yahşihan, Türkiye; erayalcigir@kku.edu.tr (M.E.A.); 243358001@kku.edu.tr (F.A.); 4 Department of Veterinary Microbiology, Department of Preclinical Sciences, Faculty of Veterinary Medicine, Kırıkkale University, 71450 Yahşihan, Türkiye; sibelkizil@kku.edu.tr; 5 Laboratory and Veterinary Health Program, Department of Veterinary Medicine, Alaca Avni Çelik Vocational School, Hitit University, 19000 Çorum, Türkiye; goksadcemilkotan@hitit.edu.tr; 6 Erzurum Regional Training and Research Hospital, 25240 Yakutiye, Türkiye

**Keywords:** sepsis, cecal ligation and puncture, *Annona muricata*, oxidative stress, antioxidant defense, chemerin, asprosin, BDNF, bacterial translocation, good health and well-being

## Abstract

Oxidative stress, immune dysregulation, and disturbed metabolic adaptation contribute to early sepsis pathophysiology. This exploratory study evaluated a commercial *Annona muricata* leaf extract in 40 male rats assigned to five groups (*n* = 8/group): healthy Control, laparotomy-only Sham, untreated cecal ligation and puncture (CLP), CLP plus a single 100 mg/kg extract dose, and CLP plus a single 200 mg/kg extract dose. Redox indices, circulating neuroendocrine and inflammatory biomarkers, bacterial translocation and peritoneal immune variables, 24 h functional measures, and multiorgan histopathology were assessed. All 40 animals reached the predefined 24 h endpoint. Relative to healthy Control and Sham animals, untreated CLP rats showed higher MDA, TOS, OSI, chemerin, asprosin, IL-1β, mesenteric lymph-node bacterial burden, peritoneal cfDNA, MPO activity, and anaerobic load, together with lower CAT, GPx, TAS, BDNF, circulating AMPK immunoreactivity, and phagocytic activity (all omnibus *p* < 0.001). The primary treatment comparison was with untreated CLP. Both extract doses shifted most continuous outcomes toward non-septic values, with the larger shift generally observed at 200 mg/kg; for example, MDA decreased from 6.92 ± 0.54 nmol/mL in untreated CLP rats to 4.15 ± 0.32 and 2.65 ± 0.21 nmol/mL at 100 and 200 mg/kg, respectively, while OSI decreased from 3.07 ± 0.28 to 1.21 ± 0.12 and 0.65 ± 0.06. Histological findings were heterogeneous and did not demonstrate uniform multiorgan protection. These data indicate short-term, dose-ordered associations between single-dose leaf-extract administration and biochemical, microbial, immune, and functional outcomes in this male-rat CLP model; they do not establish a molecular mechanism, direct antimicrobial activity, long-term survival benefit, safety, or clinical efficacy.

## 1. Introduction

Sepsis is a life-threatening syndrome in which a dysregulated host response to infection causes organ dysfunction [1,2]. Despite prompt source control, antimicrobial therapy, hemodynamic support, and organ-supportive care, sepsis remains biologically heterogeneous and can progress through overlapping phases of hyperinflammation, immune dysfunction, endothelial and microvascular injury, altered cellular energetics, and pathogen persistence [2,3,4]. Consequently, adjunctive strategies are sought to limit host-mediated injury without compromising microbial clearance; however, benefits observed in experimental models do not necessarily translate into improved clinical survival. Cecal ligation and puncture (CLP) reproduces several features of polymicrobial sepsis of abdominal origin and remains a widely used preclinical model for examining integrated host responses [5].

Redox imbalance is a central component of sepsis-associated tissue injury. Excess reactive oxygen and nitrogen species generated by activated leukocytes, dysfunctional mitochondria, and perturbed enzymatic pathways promote membrane lipid peroxidation, endothelial dysfunction, and bioenergetic failure, while endogenous antioxidant reserves are consumed or impaired [6,7,8]. Malondialdehyde (MDA) provides an index of lipid peroxidation, whereas catalase (CAT), glutathione peroxidase (GPx), total antioxidant status (TAS), total oxidant status (TOS), and the oxidative stress index (OSI) collectively describe oxidant burden and antioxidant capacity [9,10,11]. Antioxidant and anti-inflammatory compounds are therefore plausible adjunctive candidates, but redox signaling also contributes to antimicrobial host defense, and biomarker improvement alone cannot establish therapeutic efficacy. Concordant changes across redox, inflammatory, microbial, functional, and tissue-level outcomes are needed for a more balanced preclinical interpretation.

*Annona muricata* L. (soursop or graviola) leaves contain phenolic compounds, flavonoids, alkaloids, and annonaceous acetogenins, although their abundance varies markedly with cultivar, plant part, extraction method, and batch [12,13,14]. Experimental and in vitro studies have reported antioxidant and anti-inflammatory activity of leaf preparations, including reduced oxidant generation by phagocytes and attenuation of inflammatory injury [15,16,17,18]. Leaves may provide a chemically diverse source of redox-active constituents, but this diversity is also a disadvantage: composition and bioavailability are difficult to reproduce without chromatographic characterization and marker-compound quantification. Moreover, some Annonaceae acetogenins, including annonacin, inhibit mitochondrial complex I and have raised neurotoxicity and cytotoxicity concerns [19,20]. Thus, any apparent benefit of a whole-leaf preparation must be weighed against uncertain constituent exposure, possible organ toxicity, and the inability to attribute effects to a specific compound.

Sepsis also perturbs adipokine signaling, neurotrophic support, energy sensing, intestinal barrier integrity, and innate immune function. Circulating chemerin rises early in human sepsis and may reflect metabolic-inflammatory stress [21]. Asprosin is a fasting-responsive glucogenic and orexigenic adipokine [22], but its role in sepsis remains largely undefined. Brain-derived neurotrophic factor (BDNF) and AMP-activated protein kinase (AMPK) have been linked experimentally to tissue resilience and metabolic adaptation during sepsis [23,24,25]. Against this background, the present exploratory study evaluated whether a single oral dose of commercial *A. muricata* leaf extract at 100 or 200 mg/kg was associated with improved systemic redox status 24 h after CLP, a procedure that simulates polymicrobial abdominal sepsis, and whether these changes coincided with shifts in neuroendocrine-inflammatory biomarkers, bacterial translocation, peritoneal immune function, short-term physiology, and multiorgan histology.

## 2. Materials and Methods

### 2.1. Animals and Experimental Groups

A total of 40 adult male Wistar albino rats (10–12 weeks; 220–250 g) were obtained from the Experimental Animal Laboratory of Ankara Training and Research Hospital. Animals were housed in standard polycarbonate cages (four rats/cage) during acclimatization under controlled conditions (22 ± 2 °C; relative humidity 50–55%; 12 h light/dark cycle), with ad libitum access to standard commercial rodent chow and filtered tap water. After a 7-day acclimatization period and baseline body-weight measurement, *animals were allocated in a 1:1:1:1:1 ratio using a computer-generated randomization sequence. The individual animal was the experimental unit. The five groups were Control, Sham, CLP, CLP + A. muricata* 100 mg/kg, and CLP + *A. muricata* 200 mg/kg (n = 8/group).

Control group (C): Animals underwent no surgical intervention or sepsis induction and received no *A. muricata* extract. This group was used to establish healthy baseline values.

Sham group (S): Animals underwent laparotomy and cecal manipulation without ligation or puncture and received no *A. muricata* extract. This group was included to account for the effects of anesthesia, laparotomy, and surgical manipulation.

Sepsis group (CLP): Sepsis was induced using the cecal ligation and puncture procedure, and the animals received no *A. muricata* extract. This group served as the untreated septic control.

CLP + *A. muricata* 100 mg/kg group: Following CLP-induced sepsis, animals received *A. muricata* extract at a dose of 100 mg/kg by oral gavage.

CLP + *A. muricata* 200 mg/kg group: Following CLP-induced sepsis, animals received *A. muricata* extract at a dose of 200 mg/kg by oral gavage.

**Figure 1:**
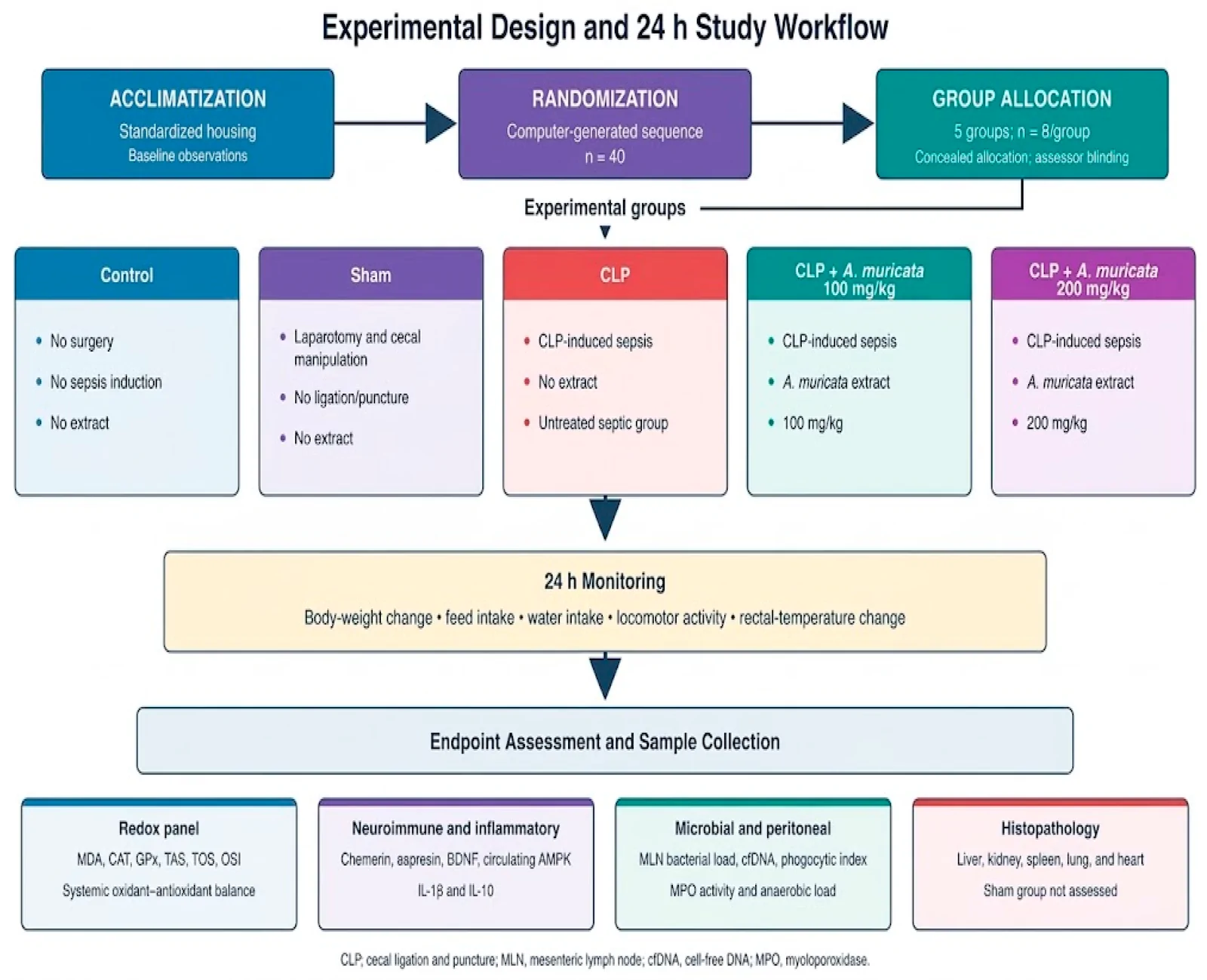
Schematic overview of the experimental design, group allocation, 24 h monitoring, and endpoint assessments in CLP-induced septic rats treated with *A. muricata* extract. CLP, cecal ligation and puncture; MLN, mesenteric lymph node; cfDNA, cell-free DNA; MPO, myeloperoxidase.

Group allocation was concealed using sequentially numbered, opaque sealed envelopes until the time of surgical intervention. Investigators conducting post-mortem biochemical assays, ELISA measurements, and microbial plate counts were blinded to group allocations. Predefined humane endpoints included severe hypothermia unresponsive to active warming, complete lack of responsiveness or mobility, and labored respiration or dyspnea. No animal reached a terminal humane endpoint, no nonterminal morbidity required additional emergency supportive care, and all 40 animals successfully completed the predefined 24 h study period without any specimen loss (n = 8 per group for all evaluated continuous outcomes). Endpoint completion within this acute timeframe is interpreted strictly as short-term survival and not as evidence of longer-term protection. All experimental procedures were reported in accordance with ARRIVE 2.0 guidelines [26] and were approved by the University of Health Sciences Ankara Training and Research Hospital Animal Experiments Local Ethics Committee (Meeting No. 0087, Decision No. 821, 16 January 2025). The experimental design, group allocation, and 24 h assessment workflow are summarized in Figure 1.

### 2.2. Cecal Ligation and Puncture Procedure

Polymicrobial sepsis was induced using the CLP model [5]. Rats were anesthetized with ketamine hydrochloride (80 mg/kg, i.p.) and xylazine hydrochloride (10 mg/kg, i.p.); tramadol hydrochloride (5 mg/kg, s.c.) was administered for postoperative analgesia. Under aseptic conditions, a 1.5–2 cm midline laparotomy was performed, the cecum was exteriorized and ligated below the ileocecal valve at approximately 50% of its length without obstructing bowel continuity, and the antimesenteric border was punctured twice through-and-through with an 18-gauge sterile needle. A small fecal droplet was extruded to confirm patency. Sham animals underwent anesthesia, laparotomy, cecal exteriorization, and closure without ligation or puncture. Immediately after surgery, all surgically manipulated animals (Sham and all CLP groups) received warm physiological saline (3 mL/100 g, s.c.) for fluid resuscitation and were maintained on heating pads at 37 °C until full recovery of the righting reflex. Sham and all CLP-operated animals followed the identical postoperative warming, monitoring, and tramadol analgesia schedule. Control animals underwent no surgical intervention or anesthesia and received oral vehicle gavage only. Each animal received only one assigned post-recovery gavage: a single extract dose (100 or 200 mg/kg) in the corresponding treatment group or a single vehicle dose in the Control, Sham, and untreated CLP groups. This single administration was performed at 10 mL/kg within 30 min after full anesthetic recovery. No repeat extract dose was given during the 24 h observation period. No antibiotics were administered. Animals were monitored every 2–4 h. At 24 h, after completion of functional observations, animals were deeply anesthetized (ketamine/xylazine, 100/10 mg/kg, i.p.) and euthanized by exsanguination through cardiac puncture for sample collection.

### 2.3. Annona muricata Extract and Treatment

The treatment groups received a commercial dry powdered *A. muricata* leaf extract (Naturalya, Ankara, Türkiye) as a single oral dose of 100 or 200 mg/kg body weight. These two exploratory doses were selected a priori to provide lower- and higher-dose comparisons informed by prior rodent research with *A. muricata* leaf preparations [16] and by feasibility at the fixed gavage volume; no formal dose-finding experiment was performed, and neither dose is presented as therapeutically optimal. The powder was freshly suspended in 0.5% carboxymethyl cellulose (CMC) in sterile physiological saline and administered once by oral gavage at 10 mL/kg. Control, Sham, and untreated CLP animals received an identical volume of vehicle at the corresponding post-recovery time point. The commercial preparation was stored in an opaque, airtight container protected from light and moisture at 4 °C until use. The available product documentation identified the material only as a whole-leaf extract; it did not provide an extraction ratio, solvent specification, chromatographic fingerprint, quantitative marker-compound content, acetogenin concentration, certificate of analysis, or batch-specific contaminant profile. Consequently, the preparation cannot be described as chemically standardized, and “standardized” refers only to the fixed administered mass per kilogram and gavage volume, not to a verified phytochemical composition. The material is therefore designated throughout as an unstandardized commercial whole-leaf preparation. This absence of batch characterization precludes attribution of the observed outcomes to a specific constituent and is treated as a major study limitation.

### 2.4. Sampling and Pre-Analytical Handling

Body weight and rectal temperature were recorded at baseline (0 h) and at 24 h post-induction. Feed and water consumption were measured cumulatively over the 24 h monitoring period, and spontaneous locomotor activity was assessed as crossings during a 5 min observation period in an open-field arena. At the terminal 24 h time point, non-fasted rats were deeply anesthetized with ketamine/xylazine (100/10 mg/kg, i.p.). Blood samples were collected via cardiac puncture into serum separator tubes, allowed to clot for 30 min at room temperature, and centrifuged at 1500× *g* for 15 min at 4 °C. Serum aliquots were immediately snap-frozen and stored at −80 °C until biochemical and immunoassay analyses without repeated freeze–thaw cycles. Peritoneal lavage was performed aseptically by instilling 5 mL of sterile ice-cold phosphate-buffered saline (PBS, pH 7.4) into the peritoneal cavity, followed by gentle massage and aspiration for microbial load, cell-free DNA (cfDNA), and macrophage phagocytic assays. Mesenteric lymph nodes (MLN) were excised under sterile conditions and transferred on ice in sterile transport broth for quantitative microbiology. Finally, visceral organs (liver, kidney, spleen, lung, and heart) were dissected; portions were fixed in 10% neutral-buffered formalin for histopathology, and remaining tissues were snap-frozen for enzymatic assays.

### 2.5. Oxidative Stress and Antioxidant Measurements

Serum samples and tissue homogenates were evaluated for systemic redox balance. Serum malondialdehyde (MDA) levels were measured using a rat-specific commercial ELISA kit (SinoGeneClon Biotech Co., Ltd., Hangzhou, China; Cat. No. SG-20889) according to the manufacturer’s protocol. Optical density was measured at 450 nm using an Epoch microplate spectrophotometer (BioTek Instruments, Inc., Winooski, VT, USA), and analyte concentrations were calculated from the corresponding standard curves. MDA results were expressed as nmol/mL. For antioxidant enzyme activities, liver tissues were homogenized in ice-cold phosphate-buffered saline (PBS, pH 7.4; 1:10 *w*/*v*) and centrifuged at 10,000× *g* for 15 min at 4 °C. Catalase (CAT) activity was determined by measuring the decomposition rate of hydrogen peroxide at 240 nm, and glutathione peroxidase (GPx) activity was measured by following NADPH oxidation at 340 nm in tissue supernatants; both enzyme activities were normalized to total tissue protein quantified by the Bradford assay and expressed as U/mg protein. Total antioxidant status (TAS; mmol Trolox Eq/L) and total oxidant status (TOS; µmol H_2_O_2_ Eq/L) were determined using commercial colorimetric assay kits (Elabscience Bionovation Inc., Houston, TX, USA; Cat. No. E-BC-K801-M for TAS, detection range: 0.23–2 mmol Trolox Equiv./L; and Cat. No. E-BC-K802-M for TOS, detection range: 2.5–100 µmol H_2_O_2_ Equiv./L) according to the manufacturer’s instructions. The oxidative stress index (OSI, arbitrary unit) was calculated as follows:

OSI (arbitrary unit) = [TOS (µmol H_2_O_2_ Eq/L)/TAS (µmol Trolox Eq/L)] × 100 = TOS/(TAS × 10)

Absorbance was measured using an Epoch microplate spectrophotometer (BioTek Instruments, Inc., Winooski, VT, USA) at 660 nm for TAS and 590 nm for TOS. All assays ere performed in duplicate with intra- and inter-assay coefficients of variation (CV) < 8%.

### 2.6. Neuroendocrine and Inflammatory Biomarkers

Serum concentrations of neuroendocrine and inflammatory biomarkers were determined in duplicate using commercial rat-specific sandwich ELISA kits according to the manufacturers’ instructions: asprosin (Cat. No. ER1944; Wuhan Fine Biotech Co., Ltd., Wuhan, Hubei, China; detection range: 62.5–4000 pg/mL, sensitivity: 37.5 pg/mL), AMPK (Cat. No. ER0730; Wuhan Fine Biotech Co., Ltd., Wuhan, Hubei, China; detection range: 3.125–200 ng/mL, sensitivity: 1.875 ng/mL), IL-1β (Cat. No. E0119Ra; Shanghai Korain Biotech Co., Ltd., Shanghai, China; detection range: 0.2–60 ng/mL, sensitivity: 0.08 ng/mL), IL-10 (Cat. No. E0108Ra; Shanghai Korain Biotech Co., Ltd., Shanghai, China; detection range: 3–900 pg/mL, sensitivity: 1.51 pg/mL), BDNF (Cat. No. SG-20200; SinoGeneClon Biotech Co., Ltd., Hangzhou, China; detection range: 3–100 ng/L, sensitivity: 1 ng/L), and chemerin (Cat. No. ER0864; Wuhan Fine Biotech Co., Ltd., Wuhan, Hubei, China; detection range: 0.156–10 ng/mL, sensitivity: 0.094 ng/mL). Optical density was measured at 450 nm (with 630 nm reference correction) using an Epoch microplate spectrophotometer (BioTek Instruments, Inc., Winooski, VT, USA). Analyte concentrations were calculated from standard curves generated using a four-parameter logistic (4-PL) curve-fitting algorithm. Intra- and inter-assay coefficients of variation were <10% for all assays. The AMPK assay measured circulating immunoreactive protein and did not quantify tissue-level enzyme phosphorylation or kinase activation.

### 2.7. Bacterial Translocation and Peritoneal Immune Measurements

Mesenteric lymph nodes (MLNs) were weighed, homogenized in sterile PBS (1:10 *w*/*v*), serially diluted, and plated in duplicate on blood agar and MacConkey agar. Plates were incubated aerobically at 37 °C for 24–48 h; counts were expressed as log_10_ CFU/g tissue with a lower limit of detection of 10 CFU/g. Culture plates exhibiting no colony growth were assigned a baseline value equal to half of the detection limit (5 CFU/g) prior to logarithmic transformation. Standard colony morphology and Gram staining were performed to confirm enteric isolates. For peritoneal microbiology, peritoneal lavage fluid was collected following standardized aseptic instillation of 5 mL ice-cold sterile PBS into the abdominal cavity, yielding consistent recovery volumes across all animals (4.2 ± 0.3 mL). Aliquots were plated in duplicate on Bacteroides Bile Esculin Agar and incubated anaerobically (<0.1% O_2_, >15% CO_2_, verified with anaerobic indicator strips) at 37 °C for 48 h; viable counts were expressed as log_10_ CFU/mL (detection limit: 10 CFU/mL).

The remaining peritoneal lavage fluid was centrifuged at 1500× *g* for 10 min at 4 °C to separate cellular elements. The resulting cell-free supernatant was collected, and cell-free DNA (cfDNA) concentrations were quantified using the Quant-iT PicoGreen dsDNA assay kit (Invitrogen, Thermo Fisher Scientific, Waltham, MA, USA; Cat. No. P7589) against standard lambda DNA calibration curves according to the manufacturer’s protocol, with results expressed as ng/µL. Peritoneal macrophages were harvested from the cell pellet using Ficoll-Paque density-gradient centrifugation, washed, and resuspended in RPMI-1640 medium. Macrophage suspensions (1 × 10^6^ cells/mL) were incubated with unopsonized zymosan particles at a fixed particle-to-cell ratio of 10:1 for 60 min at 37 °C in 5% CO_2_. Phagocytic activity was assessed by an experienced investigator blinded to experimental allocations; the phagocytic index was calculated as the percentage of macrophages that had ingested two or more particles among 200 evaluated cells. Peritoneal tissue/exudate myeloperoxidase (MPO) activity was determined using the o-dianisidine–H_2_O_2_ oxidation method at 460 nm and normalized to tissue wet weight, expressed as U/g tissue. Individual animal-level raw numerical data for all microbiologic and peritoneal variables are archived and accessible upon request. These assays characterize acute local culture burden and ex vivo immune indices; they do not indicate direct antimicrobial or bactericidal activity of the extract.

### 2.8. Functional and Physiological Outcomes

Relative body-weight change was calculated against the pre-surgical baseline [% ΔBW = ((BW_24h_ − BW_0h_)/BW_0h_) × 100]. Animals were individually housed in metabolic cages during the observation period to record cumulative feed intake (g/rat/24 h) and water consumption (mL/rat/24 h), with correction for spillage and evaporation. Spontaneous locomotor activity was evaluated once at the 24 h terminal endpoint, immediately before euthanasia, in an open-field arena (50 × 50 × 40 cm) by an observer blinded to the experimental groups, and total line crossings were recorded during a 5 min session. Animals were not habituated or trained in the arena prior to testing. Light intensity in the test room was kept uniform under standard diffuse ceiling illumination (~100–120 lux at arena floor level) to minimize novelty-induced anxiety, although lux levels were not continuously logged across trials. Because animals were not habituated and factors such as surgical stress, illness severity, and drug clearance could influence exploratory drive, these measures are strictly interpreted as short-term functional and clinical readouts rather than isolated neurobehavioral phenotypes. Rectal temperature was measured at fixed clock times using a lubricated digital rodent probe inserted to a standardized depth of 2.0 cm (ΔT = T_24h_ − T_0h_).

### 2.9. Histopathological Examination

Liver, kidney, spleen, lung, and heart tissues collected from the Control, CLP, CLP + *A. muricata* 100 mg/kg, and CLP + *A. muricata* 200 mg/kg groups (n = 8 per group; the Sham group was not evaluated histologically) were fixed in 10% neutral-buffered formalin for 48 h, processed through graded ethanol and xylene series, and embedded in paraffin wax. Sections (4 µm thick) were stained with hematoxylin and eosin (H&E) and examined under an Olympus BX53 light microscope (Olympus Corporation, Tokyo, Japan) equipped with a digital imaging system. To ensure rigorous histopathological assessment, a predefined semiquantitative scoring system was applied by a board-certified veterinary pathologist who was completely blinded to the experimental groupings. Ten randomly selected, non-overlapping microscopic fields per tissue section were systematically evaluated under a 10× objective (confirmed at 20× and 40× magnification). Three distinct histopathological lesion domains were evaluated in each organ: (i) degeneration and necrosis (e.g., hepatocyte vacuolation and necrosis, renal tubular epithelial degeneration and cast formation, myocardial myofiber degeneration and contraction bands, alveolar epithelial damage); (ii) inflammatory infiltration (e.g., focal/diffuse leukocytic infiltration in interstitial or sinusoidal areas, alveolar neutrophil extravasation, splenic red/white pulp inflammatory expansion); and (iii) vascular alterations (e.g., hyperemia, sinusoidal congestion, microvascular thrombosis, perivascular edema, capillary hemorrhage). For each animal, organ, and lesion domain, the severity of histopathological alteration was semiquantitatively categorized based on the estimated percentage of tissue involvement across the examined fields: absent (−; 0%), minimal (−/+; 1–10%), mild (+; 11–30%), mild-to-moderate (+/++; 31–50%), moderate (++; 51–70%), moderate-to-severe (++/+++; 71–90%), and severe (+++; 91–100%).

### 2.10. Statistical Analysis

Continuous data are presented as mean ± SD. Distributional and variance assumptions were evaluated from model residuals using Shapiro–Wilk and Levene tests, respectively. Group differences were analyzed by one-way ANOVA followed by Duncan’s multiple-range test; the individual animal was the experimental unit. Microbial counts were analyzed on the log10 scale. The phagocytic index was analyzed on its original percentage scale; because observed group means were not concentrated near 0% or 100%, ANOVA was retained, but no binomial model was fitted. Means with different superscript letters differ at *p* < 0.05. Omnibus *p* values are reported. Analyses used SPSS Statistics 26.0 (IBM Corp., Armonk, NY, USA). No family-wise multiplicity adjustment across the large endpoint panel was reported; results are therefore interpreted as exploratory and with emphasis on cross-domain consistency rather than isolated *p* values.

## 3. Results

### 3.1. Systemic Redox Status

CLP induced a coherent oxidant-dominant shift (Table 1). Compared with the Control and Sham groups, untreated septic rats had markedly higher MDA, TOS, and OSI values and lower CAT, GPx, and TAS values. The absence of material differences between Control and Sham groups indicates that laparotomy alone did not reproduce the magnitude of the CLP-associated redox disruption.

Both extract doses shifted the redox profile toward non-septic values. At 100 mg/kg, MDA decreased from 6.92 ± 0.54 to 4.15 ± 0.32 nmol/mL, while CAT, GPx, and TAS increased and TOS and OSI decreased relative to untreated CLP. At 200 mg/kg, MDA and OSI fell further to 2.65 ± 0.21 nmol/mL and 0.65 ± 0.06, respectively; CAT, GPx, and TAS rose to 35.1 ± 2.8 U/mg protein, 25.2 ± 2.0 U/mg protein, and 1.72 ± 0.13 mmol Trolox equivalents/L. Overall group differences were significant for all redox variables (*p* < 0.001).

### 3.2. Neuroendocrine and Inflammatory Profile

CLP produced a broad neuroendocrine-inflammatory disturbance (Table 2). Chemerin, asprosin, and IL-1β were higher in the untreated CLP group than in the two non-septic groups, whereas BDNF and circulating AMPK immunoreactivity were lower. Both extract doses produced graded shifts toward the Control and Sham values. Chemerin decreased from 58.6 ± 4.5 ng/mL in CLP to 38.2 ± 2.9 and 24.5 ± 1.8 ng/mL at 100 and 200 mg/kg, respectively. Asprosin and IL-1β followed the same direction, whereas BDNF increased from 165.4 ± 18.5 pg/mL to 265.0 ± 22.4 and 370.2 ± 28.1 pg/mL.

IL-10 showed a distinct pattern: it was lowest in untreated CLP (15.2 ± 1.4 pg/mL) but increased to 58.4 ± 4.6 and 76.2 ± 5.8 pg/mL in the two treatment groups. All omnibus group comparisons were significant (*p* < 0.001). Because tissue phosphorylation was not measured, the AMPK values describe circulating antigen concentration rather than kinase activation.

### 3.3. Bacterial Translocation and Peritoneal Immune Variables

CLP increased mesenteric lymph-node bacterial translocation and anaerobic peritoneal load, accompanied by higher peritoneal cfDNA and MPO activity and a lower macrophage phagocytic index (Table 3). Mesenteric lymph-node burden rose from 0.5 ± 0.3 log_10_ CFU/g in Control to 4.85 ± 0.42 log_10_ CFU/g in untreated CLP, while the phagocytic index fell from 82.5 ± 4.8% to 28.4 ± 2.6%.

Both extract doses improved this profile. At 200 mg/kg, bacterial translocation, cfDNA, MPO activity, and anaerobic load decreased to 1.62 ± 0.18 log_10_ CFU/g, 34.2 ± 2.8 ng/µL, 3.45 ± 0.28 U/g tissue, and 1.12 ± 0.15 log_10_ CFU/mL, respectively; the phagocytic index increased to 66.5 ± 4.1%. All group differences were significant (*p* < 0.001).

### 3.4. Short-Term Functional and Physiological Measures

All 40 animals reached the 24 h endpoint, and Table 4 reports n = 8/group. Untreated CLP animals showed the largest weight loss, lowest intake and spontaneous crossings, and greatest decrease in rectal temperature. Both extract-treated groups showed graded shifts toward non-septic values across these short-term measures; complete descriptive and inferential results are provided in Table 4.

### 3.5. Histopathological Findings

Histopathological findings were heterogeneous across organs (Table 5; n = 8/group evaluated in Control, CLP, CLP + 100 mg/kg, and CLP + 200 mg/kg). Low-grade myocardial and pulmonary vascular findings were also recorded in Control animals. In the absence of Sham histology, serial sections, or lesion-specific ancillary staining, these background findings cannot be distinguished confidently among spontaneous change, tissue processing/sampling artifact, and semiquantitative scoring variability.

**Figure 2:**
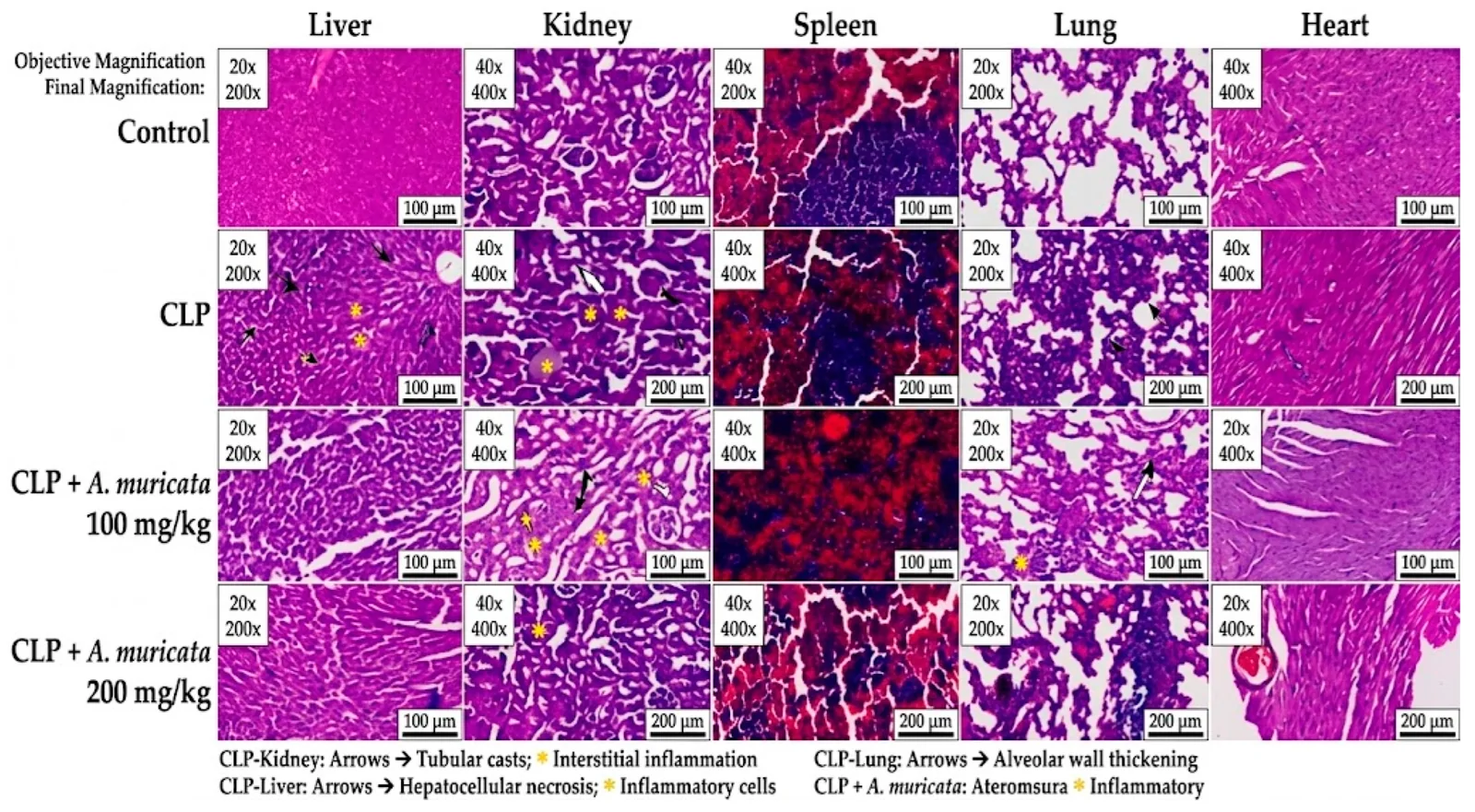
Representative H&E-stained sections of liver, kidney, spleen, lung, and heart from Control, CLP, CLP + *A. muricata* 100 mg/kg, and CLP + *A. muricata* 200 mg/kg groups. Columns indicate organs and rows indicate experimental groups. Black arrows indicate structural degeneration and necrosis (e.g., hepatocyte necrosis, renal tubular casts, alveolar wall thickening), and yellow asterisks indicate vascular congestion and leukocytic infiltration. Images were captured at 200× and 400× final magnification (objective magnification: 20× and 40×). Calibrated scale bars = 100 µm or 200 µm as indicated on individual panels. CLP, cecal ligation and puncture; H&E, hematoxylin and eosin.

At 100 mg/kg, hepatic degeneration/necrosis was graded lower than in untreated CLP (+ versus +/++), but selected renal, pulmonary, and myocardial changes persisted. At 200 mg/kg, renal and pulmonary findings were generally minimal; however, hepatic degeneration/necrosis remained +/++, and mild myocardial degeneration and vascular alteration persisted. Thus, histology did not show a uniform dose-related improvement despite the graded biochemical and functional pattern. Representative H&E-stained sections from the evaluated organs and groups are shown in Figure 2.

## 4. Discussion

The principal finding was a broad, internally consistent association between *A. muricata* extract administration and short-term redox, inflammatory, microbial, and functional measures after CLP. Untreated CLP animals showed lipid peroxidation, depleted antioxidant defenses, inflammatory activation, bacterial translocation, impaired peritoneal phagocytosis, hypothermia, reduced intake, weight loss, and reduced activity. Both doses shifted most continuous endpoints toward non-septic values, generally more strongly at 200 mg/kg. Histopathology was less consistent, particularly in the liver. Thus, the experiment supports acute exploratory effects in male rats, not complete multiorgan protection or a causal molecular mechanism.

Redox disruption is biologically central to sepsis: activated leukocytes, mitochondrial dysfunction, xanthine oxidase, and uncoupled nitric-oxide pathways increase oxidant generation, while enzymatic and non-enzymatic defenses are consumed or impaired [6,7]. In this CLP experiment, higher MDA, TOS, and OSI together with lower CAT, GPx, and TAS formed a coherent acute redox pattern, and both extract doses shifted these measures toward Control and Sham values. This direction is compatible with cell-based and experimental evidence: Saraiva et al. studied a polyphenol-rich *A. muricata* leaf fraction in neutrophils, macrophages, and an experimental lung-injury model and reported attenuation of oxidative and inflammatory responses [17], whereas George et al. demonstrated antioxidant and DNA-protective activity alongside HPLC characterization of *A. muricata* extracts [18]. Those studies differ from the present polymicrobial CLP design in extract preparation, biological model, and endpoint timing; therefore, they support biological plausibility but cannot identify the active constituents or prove transferability to this batch. Nrf2 activation, NF-κB inhibition, and direct radical scavenging were not measured.

A cautious working model can be proposed from the cross-domain pattern, but it remains a hypothesis rather than a demonstrated pathway. Leaf-derived phenolics and flavonoids could reduce reactive-species propagation or influence redox-sensitive inflammatory signaling, thereby limiting lipid peroxidation and secondary leukocyte activation; preserved CAT and GPx activity could then support peroxide removal, while lower inflammatory stress could help maintain phagocytic function, barrier integrity, temperature, and intake [8,14,17]. The graded biochemical pattern and simultaneous reductions in IL-1β, MPO, cfDNA, and culture burden are compatible with this sequence. Recent CLP studies of other antioxidant compounds have likewise reported parallel redox and inflammatory improvements, supporting the general biological plausibility of redox-directed modulation rather than a leaf-extract-specific mechanism [27]. However, the present experiment did not quantify extract constituents, tissue drug exposure, mitochondrial respiration, Nrf2, NF-κB, inflammasome activation, receptor signaling, or organism-specific killing. Because annonaceous acetogenins can themselves impair mitochondrial complex I [19], the same chemically undefined preparation could contain constituents with opposing biological actions. The data therefore cannot distinguish direct antioxidant action, indirect preservation of host physiology, altered absorption or metabolism, or nonspecific effects of lower illness severity.

Pro- and anti-inflammatory programs overlap during sepsis, so IL-10 elevation alone is not necessarily beneficial [3]. In the present study, lower IL-1β and higher IL-10 in treated animals occurred alongside reduced MPO activity and cfDNA, improved ex vivo phagocytosis, lower culture burden, and less severe short-term physiological disturbance. These anti-inflammatory and tissue-protective patterns are conceptually consistent with earlier investigations into *A. muricata*, although experimental paradigms differ substantially. Specifically, de Sousa et al. [16] evaluated an ethanolic leaf extract in classic rodent models of acute chemical nociception and local inflammation (acetic acid-induced abdominal contortions, formalin testing, and carrageenan-induced paw edema). They demonstrated significant peripheral antinociception and suppression of localized exudation and leukocyte recruitment, which they largely attributed to the inhibition of cyclooxygenase pathways and reduced local release of pro-inflammatory mediators. In a complementary mechanistic study, Saraiva et al. [17] investigated a polyphenol-rich leaf fraction using both in vitro murine immune cells (isolated neutrophils and peritoneal macrophages) and an in vivo lipopolysaccharide (LPS)-induced acute lung injury (ALI) mouse model. They reported that the fraction inhibited reactive oxygen species (ROS) and nitric oxide generation, attenuated neutrophil chemotaxis, and reduced TNF-α and IL-1β levels in bronchoalveolar lavage fluid by modulating the NF-κB pathway. While both earlier studies corroborate the intrinsic capacity of *A. muricata* constituents to downregulate acute inflammatory cascades and dampen neutrophilic activity, critical distinctions exist when compared to the present work. The models employed by de Sousa et al. (sterile paw edema/writhing) and Saraiva et al. (sterile endotoxemia via purified LPS) represent transient, non-infectious inflammatory insults characterized by an isolated pro-inflammatory trigger. In contrast, our study utilized the cecal ligation and puncture (CLP) model, which establishes a persistent, polymicrobial intra-abdominal infectious focus driving simultaneous hyperinflammation, immune paralysis, and severe multi-organ dysfunction. Furthermore, whereas Saraiva et al. investigated a sub-fractionated polyphenol extract under parenteral/local delivery, our protocol administered an unstandardized whole-leaf aqueous/organic commercial extract via the oral route following sepsis induction. Despite these differences in model etiology, extract formulation, and pharmacokinetics, the common observation across all three studies is the marked blunting of neutrophil-mediated inflammatory stress (evidenced here by reduced systemic IL-1β and peritoneal MPO activity). However, because CLP involves living, replicating polymicrobial pathogens rather than isolated chemical or sterile endotoxic stimuli, the improved immune and cytokine balance observed in our model likely reflects indirect systemic preservation and cellular resilience rather than a single isolated anti-inflammatory pathway.

The altered profiles of chemerin and asprosin broaden the metabolic interpretation of our findings, though direct translational comparisons require cautious evaluation. Circulating adipokines serve as critical integrators of energy metabolism, systemic inflammation, and innate immune defense. Karampela et al. [21] conducted a prospective observational clinical study in adult intensive care unit (ICU) patients, systematically monitoring circulating chemerin levels and kinetics over a 28-day period. They demonstrated that serum chemerin was significantly elevated at ICU admission in patients with sepsis and septic shock compared to healthy and critically ill non-septic controls, and that sustained high chemerin levels correlated with elevated APACHE II and SOFA severity scores and predicted poor clinical outcomes. In our preclinical interventional study, untreated CLP rats exhibited a similarly robust surge in circulating chemerin at 24 h, whereas *A. muricata* administration produced a dose-dependent reduction toward non-septic baseline levels. However, substantial design distinctions separate these investigations. The cohort examined by Karampela et al. comprised heterogeneous human patients with varied sepsis etiologies, baseline comorbidities, and extensive supportive therapy, with biomarker kinetics tracked over several weeks. In contrast, our study evaluated a standardized, acute surgical sepsis insult in healthy adult male rats at a single 24 h endpoint following oral phytotherapeutic treatment. Furthermore, because we did not quantify tissue-level chemerin receptor (CMKLR1) expression, downstream intracellular pathways, or adipocyte biology, our findings demonstrate an exploratory systemic association rather than direct receptor-mediated modulation.

The patterns observed for circulating BDNF and AMPK similarly warrant a detailed comparative evaluation against published sepsis literature. In an experimental investigation of septic cardiomyopathy, Zeng et al. [23] employed a rat CLP model to assess the cardioprotective potential of exogenous recombinant BDNF administration. Their findings demonstrated that direct systemic administration of BDNF preserved cardiac contractility, suppressed cardiomyocyte apoptosis, and attenuated myocardial inflammation via the activation of endothelial nitric oxide synthase (eNOS) and subsequent nitric oxide (NO) pathway signaling. In parallel, preclinical studies focusing on cellular energy homeostasis have established AMP-activated protein kinase (AMPK) as a master regulator of metabolic adaptation during severe systemic infection. Escobar et al. [24] utilized a mouse CLP model and demonstrated that pharmacological pre-treatment or early post-treatment with AMPK activators (such as AICAR or metformin) mitigated acute kidney and liver injury, inhibited systemic pro-inflammatory cytokine release, and maintained cellular integrity by blocking NF-κB nuclear translocation. Furthermore, Jin et al. [25] demonstrated in both murine CLP and endotoxemia models that genetic or pharmacological enhancement of AMPK activity restored mitochondrial biogenesis, preserved fatty acid oxidation, and significantly improved long-term survival by sustaining cellular metabolic fitness.

While these investigations provide biological plausibility for the protective involvement of BDNF and AMPK pathways during septic shock, essential distinctions from our experimental design must be highlighted. Both Zeng et al. and the AMPK intervention studies evaluated specific, targeted pharmacological agonists or direct recombinant peptides aimed at stimulating defined intracellular signaling cascades. In contrast, the present study did not administer pathway-specific agonists; rather, we observed a dose-dependent recovery of endogenous circulating BDNF and AMPK-immunoreactive protein levels following oral administration of a whole *A. muricata* leaf extract in septic rats. Crucially, our immunoassay protocol measured circulating immunoreactive AMPK protein in serum rather than intracellular enzyme phosphorylation (p-AMPK at Thr172) [28] or downstream target activation in vital organs (such as liver or myocardium). Therefore, while Zeng et al. [23] and Jin et al. [25] directly established causal, pathway-specific protection, our circulating data cannot confirm active myocardial eNOS signaling or intracellular AMPK pathway kinase activation. Instead, the elevation of circulating BDNF and preservation of serum AMPK immunoreactivity in *A. muricata*-treated groups are best interpreted as exploratory biomarkers reflecting an attenuated state of systemic metabolic collapse and preserved neuroendocrine reserve.

The culture and peritoneal findings were compatible with improved containment of the abdominal infectious challenge. In polymicrobial peritonitis, progressive breakdown of the gut mucosal barrier facilitates bacterial translocation across the mesenteric lymph node (MLN) axis into the systemic circulation, a pivotal driver of secondary sepsis-induced organ failure reviewed comprehensively by Potruch et al. [29]. Concurrently, elevated circulating cell-free DNA (cfDNA) and myeloperoxidase (MPO) activity serve as sensitive markers of active infection and innate immune activation. Hamaguchi et al. [30] investigated cfDNA kinetics in a murine CLP sepsis model and revealed that extracellular DNA fragments originate both from host tissue apoptosis/necrosis and from neutrophil extracellular trap (NET) formation driven by acute bacteremia. Furthermore, clinical investigations by Saukkonen et al. [31] in severe sepsis patients and Maruchi et al. [32] in septic shock cohorts demonstrated that high circulating cfDNA and elevated plasma MPO–DNA complexes are strongly correlated with illness severity, multi-organ dysfunction, and 28-day mortality.

While these studies establish the pathophysiological relevance of cfDNA and MPO in predicting adverse clinical trajectories, their focus differed fundamentally from our interventional design. The clinical cohorts evaluated by Saukkonen et al. and Maruchi et al. were observational human studies assessing systemic biomarkers at intensive care unit admission, whereas Hamaguchi et al. focused on dissecting the cellular origins of cfDNA in mice without therapeutic modulation. In contrast, the present rat CLP study implemented an interventional approach testing *A. muricata* extract, demonstrating concurrent reductions in MLN bacterial burden, peritoneal cfDNA concentrations, and exudate MPO activity alongside a preserved ex vivo macrophage phagocytic index. However, our experimental protocol did not include a NET-specific immunoassay (such as citrullinated histone H3 quantification), organism-resolved taxonomic profiling, or sequence-based source attribution of cfDNA to distinguish host versus microbial origin. Consequently, while lower MLN and peritoneal burdens coincide with reduced inflammatory enzymes, these outcomes reflect enhanced local pathogen containment and attenuated neutrophilic burst rather than a verified direct bactericidal mechanism.

Reduced spontaneous locomotion accompanied by profound hypothermia is a well-characterized functional hallmark of acute polymicrobial sepsis in rodents. Brooks et al. [33] systematically documented the temporal progression of CLP-induced sepsis in adult rats, demonstrating that severe intra-abdominal infection consistently triggers a marked, progressive decline in core body temperature and exploratory behavior within 12 to 24 h post-induction, serving as a reliable surrogate for systemic illness severity and multi-organ decompensation.

Our current observations align closely with the phenotypic deterioration described by Brooks et al., as untreated septic rats exhibited severe hypothermia (−2.85 ± 0.25 °C) and a drastic drop in spontaneous open-field line crossings alongside significant body-weight loss. Administration of *A. muricata* extract markedly attenuated this functional decline in a dose-dependent manner. Nonetheless, important experimental differences must be noted. Brooks et al. monitored animals longitudinally across extended time courses and integrated continuous telemetry and extensive clinicopathological staging to characterize spontaneous disease progression without therapeutic intervention. In contrast, our study evaluated functional readouts at a single terminal 24 h cross-section following acute oral extract administration. Furthermore, because our animals were not pre-habituated to the open-field apparatus, line crossings reflect a composite of acute illness, arousal, and novelty-induced exploratory drive rather than a validated, isolated neurobehavioral trait.

The marked improvements in body weight retention, cumulative oral intake, rectal temperature, and open-field crossings observed in the *A. muricata*-treated groups indicate an attenuated clinical deterioration over 24 h; however, these readouts must be interpreted with explicit consideration of potential perioperative pharmacological confounders. All surgically managed groups (Sham, CLP, and treatment arms) received identical perioperative regimens comprising ketamine/xylazine anesthesia and single-dose subcutaneous tramadol analgesia. Both ketamine (an NMDA receptor antagonist) and xylazine (a potent α2-adrenoceptor agonist) induce well-documented, dose-dependent central nervous system depression, muscle relaxation, peripheral vasoconstriction, transient hypothermia, and transient suppression of swallowing and alimentary drive. Although their direct plasma elimination half-lives in rodents are typically measured in hours, residual neurobehavioral effects, altered autonomic tone, or post-anesthetic sedation can persist into the early recovery window. Similarly, while tramadol was administered to fulfill mandatory ethical requirements for post-laparotomy pain control, its central μ-opioid receptor agonism and monoamine reuptake inhibition are recognized causes of transient motor hypoactivity, altered exploratory drive, and decreased gastrointestinal motility.

Importantly, anesthesia recovery times and functional testing schedules warrant careful procedural scrutiny. While active warming on heating pads was maintained until purposeful righting reflex was regained, individual recovery latencies were not logged as formal quantitative continuous outcomes. In severe polymicrobial sepsis, impaired hepatic microperfusion, evolving acute renal failure, and hypothermia may delay the metabolic clearance of ketamine, xylazine, and tramadol relative to non-septic Sham controls. Consequently, the profound immobility, hypophagia, and adipsia recorded in untreated CLP animals likely reflect a composite outcome: sepsis-induced encephalopathy and systemic prostration exacerbated by delayed drug clearance. Conversely, while all open-field testing was conducted within a standardized terminal window immediately prior to 24 h euthanasia, individual trials were necessarily performed sequentially rather than strictly simultaneously. Because Sham animals underwent identical surgical and anesthetic exposure yet maintained normal food/water intake, preserved body temperature, and high exploratory locomotion, anesthesia and analgesia alone cannot account for the severe functional failure seen in untreated CLP rats. Nevertheless, because our protocol evaluated a single 24 h endpoint without serialized recovery tracking, habituation trials, or pharmacokinetic monitoring, the improved functional scores in the extract-treated cohorts should be characterized as composite short-term functional and constitutional improvements rather than an isolated, opioid-independent neurobehavioral phenotype.

Safety considerations and thorough phytochemical characterization are critical prerequisites before any therapeutic extrapolation of *A. muricata* can be considered. Members of the Annonaceae family naturally contain annonaceous acetogenins (such as annonacin), which have been implicated in neurodegenerative and cytotoxic processes. In an experimental toxicological study, Höllerhage et al. [19] demonstrated that commercial dietary supplements and extracts derived from Annonaceae species, including *A. muricata*, contained substantial quantities of neurotoxic acetogenins that caused selective dopaminergic neuronal cell death in primary rat mesencephalic cultures through the potent inhibition of mitochondrial complex I (NADH dehydrogenase). In a related safety profile evaluation, Ferreira et al. [20] assessed *A. muricata* seed extracts using both in vitro micronucleus assays and in silico toxicity modeling, reporting observable genotoxicity, cellular proliferation arrest, and predicted metabolic liabilities tied to specific lipophilic fractions.

These published toxicological warnings underscore the necessity for caution when evaluating our present findings. While Höllerhage et al. focused on direct neurotoxicity following chronic or in vitro exposure to acetogenin-dense preparations, and Ferreira et al. evaluated seed-derived material, our protocol administered an acute, single-dose regimen of a leaf extract over a limited 24 h observation period. In our study, animals exhibited improved spontaneous locomotion and no overt mortality over 24 h; however, this short observation window cannot assess cumulative neurotoxicity, mitochondrial complex I suppression in vivo, or long-term organ safety. Because our administered commercial extract lacked certified batch-specific quantification of residual acetogenins or contaminant profiling, potential subclinical toxicities cannot be ruled out. Consequently, prior to establishing safety or therapeutic claims, rigorous chemical fingerprinting and formal, chronic sub-acute toxicological screening remain essential.

### Strengths and Limitations

A strength is the integrated endpoint panel spanning redox variables, cytokines, adipokines, BDNF, circulating AMPK immunoreactivity, culture burden, peritoneal immune indices, short-term physiology, and histology. Control–Sham agreement across continuous endpoints helps separate the CLP insult from laparotomy, although Sham histology was unavailable. Limitations include n = 8/group, a single 24 h endpoint, male animals only, two doses, no pharmacological positive control, no long-term survival analysis, no organ-function chemistry, no pharmacokinetics, and no dedicated tolerability study. All animals reached 24 h, but this does not inform later survival. Extract identity, batch composition, chemical exposure, and contaminants were not adequately documented in the submitted materials; therefore, reproducibility and attribution to specific constituents remain unresolved. Open-field activity lacked prior habituation and documented illumination, and perioperative ketamine/xylazine and tramadol are potential confounders. The large endpoint panel lacked a family-wise multiplicity strategy. Circulating AMPK does not establish pathway activation; cfDNA was not source resolved; direct antimicrobial activity was not tested; and histology lacked Sham tissue and animal-level ordinal inference. These limitations restrict the conclusions to acute exploratory associations in this male-rat CLP model.

## 5. Conclusions

In male rats evaluated 24 h after CLP, *A. muricata* leaf-extract administration was associated with graded shifts toward non-septic values across systemic redox indices, selected circulating biomarkers, culture burden, peritoneal immune measures, and short-term functional measures. Histopathological findings were partial and organ-specific, with persistent hepatic injury and low-grade background findings in Control tissues. These data justify further study using a chemically characterized batch, verified assay reporting, positive-control and tolerability arms, both sexes, organ-function tests, pharmacokinetics, pathway-level measurements, and longer follow-up. They do not establish a specific molecular pathway, direct antibacterial action, multiorgan protection, survival benefit, long-term safety, or clinical efficacy.

## Figures and Tables

**Table 1:** Effects of *Annona muricata* extract on serum redox status and hepatic antioxidant defense in CLP-induced sepsis.

Treatment Group	MDA (nmol/mL)	CAT (U/mg Protein)	GPx (U/mg Protein)	TAS (mmol Trolox Eq/L)	TOS (µmol H_2_O_2_ Eq/L)	OSI (Arbitrary Unit)
Control (n = 8)	1.68 ± 0.15 ^a^	45.2 ± 3.8 ^d^	32.4 ± 2.6 ^d^	2.10 ± 0.15 ^d^	6.1 ± 0.5 ^a^	0.29 ± 0.03 ^a^
Sham (n = 8)	1.75 ± 0.18 ^a^	43.8 ± 3.5 ^d^	31.8 ± 2.4 ^d^	2.05 ± 0.14 ^d^	6.4 ± 0.6 ^a^	0.31 ± 0.04 ^a^
CLP (n = 8)	6.92 ± 0.54 ^d^	15.6 ± 1.8 ^a^	11.2 ± 1.1 ^a^	0.82 ± 0.08 ^a^	25.2 ± 2.1 ^d^	3.07 ± 0.28 ^d^
CLP + 100 mg/kg (n = 8)	4.15 ± 0.32 ^c^	26.4 ± 2.2 ^b^	19.5 ± 1.6 ^b^	1.35 ± 0.11 ^b^	16.4 ± 1.3 ^c^	1.21 ± 0.12 ^c^
CLP + 200 mg/kg (n = 8)	2.65 ± 0.21 ^b^	35.1 ± 2.8 ^c^	25.2 ± 2.0 ^c^	1.72 ± 0.13 ^c^	11.2 ± 0.9 ^b^	0.65 ± 0.06 ^b^
Overall *p*-value	<0.001	<0.001	<0.001	<0.001	<0.001	<0.001

Data are mean ± SD. Within a column, means with different superscript letters differ significantly (*p* < 0.05; one-way ANOVA with Duncan’s multiple range test). CLP, cecal ligation and puncture; MDA, malondialdehyde; CAT, catalase; GPx, glutathione peroxidase; TAS, total antioxidant status; TOS, total oxidant status; OSI, oxidative stress index [TOS/(TAS × 10)].

**Table 2:** Neuroendocrine, adipokine-related, and inflammatory biomarkers across experimental groups.

Treatment Group	Chemerin (ng/mL)	Asprosin (ng/mL)	BDNF (pg/mL)	AMPK-IR (pg/mL)	IL-1β (pg/mL)	IL-10 (pg/mL)
Control (n = 8)	12.4 ± 1.1 ^a^	2.15 ± 0.18 ^a^	480.5 ± 35.2 ^d^	8.5 ± 0.7 ^d^	18.2 ± 1.5 ^a^	42.1 ± 3.5 ^b^
Sham (n = 8)	12.8 ± 1.2 ^a^	2.20 ± 0.21 ^a^	472.1 ± 32.8 ^d^	8.2 ± 0.6 ^d^	19.0 ± 1.6 ^a^	40.8 ± 3.2 ^b^
CLP (n = 8)	58.6 ± 4.5 ^d^	8.45 ± 0.65 ^d^	165.4 ± 18.5 ^a^	2.1 ± 0.3 ^a^	165.8 ± 12.4 ^d^	15.2 ± 1.4 ^a^
CLP + 100 mg/kg (n = 8)	38.2 ± 2.9 ^c^	5.42 ± 0.42 ^c^	265.0 ± 22.4 ^b^	4.2 ± 0.4 ^b^	98.2 ± 7.8 ^c^	58.4 ± 4.6 ^c^
CLP + 200 mg/kg (n = 8)	24.5 ± 1.8 ^b^	3.68 ± 0.29 ^b^	370.2 ± 28.1 ^c^	6.4 ± 0.5 ^c^	52.5 ± 4.1 ^b^	76.2 ± 5.8 ^d^
Overall *p*-value	<0.001	<0.001	<0.001	<0.001	<0.001	<0.001

Data are mean ± SD. Within a column, means with different superscript letters differ significantly (*p* < 0.05; one-way ANOVA with Duncan’s multiple range test). BDNF, brain-derived neurotrophic factor; AMPK, AMP-activated protein kinase; IL, interleukin.

**Table 3:** Bacterial translocation and peritoneal immune-related outcomes.

Treatment Group	MLN Bacterial Translocation (log_10_ CFU/g)	Peritoneal cfDNA (ng/µL)	Phagocytic Index (%)	Peritoneal MPO (U/g Tissue)	Anaerobic Load (log_10_ CFU/mL)
Control (n = 8)	0.5 ± 0.3 ^a^	12.4 ± 1.2 ^a^	82.5 ± 4.8 ^d^	1.12 ± 0.10 ^a^	0.7 ± 0.2 ^a^
Sham (n = 8)	0.7 ± 0.2 ^a^	13.1 ± 1.4 ^a^	80.1 ± 4.2 ^d^	1.18 ± 0.12 ^a^	0.6 ± 0.3 ^a^
CLP (n = 8)	4.85 ± 0.42 ^d^	88.6 ± 6.8 ^d^	28.4 ± 2.6 ^a^	8.95 ± 0.72 ^d^	3.92 ± 0.38 ^d^
CLP + 100 mg/kg (n = 8)	2.85 ± 0.28 ^c^	58.4 ± 4.5 ^c^	48.2 ± 3.5 ^b^	5.82 ± 0.45 ^c^	2.25 ± 0.20 ^c^
CLP + 200 mg/kg (n = 8)	1.62 ± 0.18 ^b^	34.2 ± 2.8 ^b^	66.5 ± 4.1 ^c^	3.45 ± 0.28 ^b^	1.12 ± 0.15 ^b^
Overall *p*-value	<0.001	<0.001	<0.001	<0.001	<0.001

Data are mean ± SD. Within a column, means with different superscript letters differ significantly (*p* < 0.05; one-way ANOVA with Duncan’s multiple range test). MLN, mesenteric lymph node; cfDNA, cell-free DNA; MPO, myeloperoxidase; CFU, colony-forming unit.

**Table 4:** Short-term functional and physiological outcomes.

Treatment Group	Body-Weight Change (%)	Feed Intake (g/Rat/24 h)	Water Intake (mL/Rat/24 h)	Locomotor Activity (Crossings/5 min)	Rectal ΔT (°C at 24 h)
Control (n = 8)	+1.85 ± 0.22 ^e^	22.4 ± 1.5 ^d^	28.5 ± 2.1 ^d^	48.2 ± 3.5 ^d^	−0.12 ± 0.04 ^d^
Sham (n = 8)	+1.20 ± 0.18 ^d^	21.8 ± 1.4 ^d^	27.2 ± 1.9 ^d^	46.5 ± 3.2 ^d^	−0.18 ± 0.05 ^d^
CLP (n = 8)	−7.85 ± 0.62 ^a^	4.2 ± 0.5 ^a^	8.1 ± 0.8 ^a^	8.4 ± 1.1 ^a^	−2.85 ± 0.25 ^a^
CLP + 100 mg/kg (n = 8)	−4.65 ± 0.38 ^b^	10.2 ± 0.8 ^b^	14.8 ± 1.1 ^b^	20.2 ± 1.8 ^b^	−1.82 ± 0.16 ^b^
CLP + 200 mg/kg (n = 8)	−2.45 ± 0.22 ^c^	15.6 ± 1.1 ^c^	20.4 ± 1.5 ^c^	34.1 ± 2.5 ^c^	−1.15 ± 0.10 ^c^
Overall *p*-value	<0.001	<0.001	<0.001	<0.001	<0.001

Data are mean ± SD. Within a column, means with different superscript letters differ significantly (*p* < 0.05; one-way ANOVA with Duncan’s multiple range test). Body-weight change was calculated relative to the pre-surgical baseline; rectal temperature change was ΔT = T_24h_ − T_0h_.

**Table 5:** Semiquantitative histopathological findings in the evaluated groups.

Organ	Histopathological Alteration	Control (n = 8)	CLP (n = 8)	CLP + 100 mg/kg (n = 8)	CLP + 200 mg/kg (n = 8)
Liver	Degeneration/necrosis	−/+	+/++	+	+/++
	Inflammation	−	−	−	−
	Vascular alterations	−/+	−/+	+	+
Kidney	Degeneration/necrosis	−/+	−/+	+	−/+
	Inflammation	−	−	−	−
	Vascular alterations	−/+	−/+	+	−/+
Spleen	Degeneration/necrosis	−	−	−	−
	Inflammation	−/+	+	+	−/+
	Vascular alterations	−/+	−/+	−/+	−/+
Lung	Degeneration/necrosis	−/+	−	−	−
	Inflammation	−/+	+	+	−/+
	Vascular alterations	+	−/+	+	−/+
Heart	Degeneration/necrosis	+	+	+	+
	Inflammation	−	−	−/+	−
	Vascular alterations	+	−/+	+	+

Histopathological alterations were graded by tissue involvement: −, absent (0%); −/+, minimal (1–10%); +, mild (11–30%); +/++, mild-to-moderate (31–50%). The Sham group was not included in histopathological assessment. CLP, cecal ligation and puncture.

## Data Availability

The data supporting the findings of this study are available from the corresponding author upon reasonable request.

## Abbreviations

AMPK, AMP-activated protein kinase; BDNF, brain-derived neurotrophic factor; CAT, catalase; cfDNA, cell-free DNA; CFU, colony-forming unit; CLP, cecal ligation and puncture; GPx, glutathione peroxidase; H&E, hematoxylin and eosin; IL, interleukin; MDA, malondialdehyde; MLN, mesenteric lymph node; MPO, myeloperoxidase; OSI, oxidative stress index; TAS, total antioxidant status; TOS, total oxidant status.
